# Thermally Drawn CNT-Based Hybrid Nanocomposite Fiber for Electrochemical Sensing

**DOI:** 10.3390/bios12080559

**Published:** 2022-07-24

**Authors:** Rino Nishimoto, Yuichi Sato, Jingxuan Wu, Tomoki Saizaki, Mahiro Kubo, Mengyun Wang, Hiroya Abe, Inès Richard, Tatsuo Yoshinobu, Fabien Sorin, Yuanyuan Guo

**Affiliations:** 1 Graduate School of Engineering, Tohoku University, Sendai 980-8579, Japan; rino.nishimoto.p6@dc.tohoku.ac.jp (R.N.); wu.jingxuan.q3@dc.tohoku.ac.jp (J.W.); wangmy1227@gmail.com (M.W.); hiroya.abe.c4@tohoku.ac.jp (H.A.); tatsuo.yoshinobu.a1@tohoku.ac.jp (T.Y.); 2 Frontier Research Institute for Interdisciplinary Sciences (FRIS), Tohoku University, Sendai 980-0845, Japan; yuichi.sato.c2@tohoku.ac.jp; 3 School of Engineering, Tohoku University, Sendai 980-8579, Japan; saizaki.tomoki.p4@dc.tohoku.ac.jp (T.S.); kubo.mahiro.t1@dc.tohoku.ac.jp (M.K.); 4 Institute of Materials, Ecole Polytechnique Fédérale de Lausanne (EPFL), 1015 Lausanne, Switzerland; ines.richard@outlook.com (I.R.); fabien.sorin@epfl.ch (F.S.); 5 Graduate School of Biomedical Engineering, Tohoku University, Sendai 980-8579, Japan; 6 Graduate School of Medicine, Tohoku University, Sendai 980-8575, Japan

**Keywords:** carbon nanotube (CNT), polymer composite, thermal drawing, fibers, electrochemical sensing

## Abstract

Nowadays, bioelectronic devices are evolving from rigid to flexible materials and substrates, among which thermally-drawn-fiber-based bioelectronics represent promising technologies thanks to their inherent flexibility and seamless integration of multi-functionalities. However, electrochemical sensing within fibers remains a poorly explored area, as it imposes new demands for material properties—both the electrochemical sensitivity and the thermomechanical compatibility with the fiber drawing process. Here, we designed and fabricated microelectrode fibers made of carbon nanotube (CNT)-based hybrid nanocomposites and further evaluated their detailed electrochemical sensing performances. Carbon-black-impregnated polyethylene (CB-CPE) was chosen as the base material, into which CNT was loaded homogeneously in a concentration range of 3.8 to 10 wt%. First, electrical impedance characterization of CNT nanocomposites showed a remarkable decrease of the resistance with the increase in CNT loading ratio, suggesting that CNTs notably increased the effective electrical current pathways inside the composites. In addition, the proof-of-principle performance of fiber-based microelectrodes was characterized for the detection of ferrocenemethanol (FcMeOH) and dopamine (DA), exhibiting an ultra-high sensitivity. Additionally, we further examined the long-term stability of such composite-based electrode in exposure to the aqueous environment, mimicking the in vivo or in vitro settings. Later, we functionalized the surface of the microelectrode fiber with ion-sensitive membranes (ISM) for the selective sensing of Na${}^{+}$ ions. The miniature fiber-based electrochemical sensor developed here holds great potential for standalone point-of-care sensing applications. In the future, taking full advantage of the thermal drawing process, the electrical, optical, chemical, and electrochemical modalities can be all integrated together within a thin strand of fiber. This single fiber can be useful for fundamental multi-mechanistic studies for biological applications and the weaved fibers can be further applied for daily health monitoring as functional textiles.

## 1. Introduction

In recent decades, we have witnessed a booming development of wearable electronics that can be seamlessly integrated into our daily life for health monitoring applications. The constant interest in emerging wearable electronics promotes the development of soft, flexible and multifunctional fibers and textiles [1]. Among such efforts, the thermal drawing process that has been conventionally used in the telecommunication industry became a promising mass-fabrication method to produce flexible polymer-based multifunctional fibers and textiles [2,3]. For example, fiber-based pressure sensors have been developed and woven into functional textiles for large-scale pressure monitoring [4]. Fiber-based neural probes have shown their usefulness in studying brain functionalities across optical, electrical, and chemical modalities [2]. Novel functional materials, such as metallic glasses, have also been successfully integrated in the thin strand of fibers as high-performance neural probes [5]. Thermally-drawn flexible fibers with tailored surface textures, chemistry and geometry have been developed as neural scaffolds for nerve regeneration [6,7]. These show the usefulness of such multifunctional fibers for fundamental biological studies as well as daily health monitoring applications. Among explored functionalities, electrochemical sensing, which offers robustness and simplicity in detection across various compounds [8], still remains poorly investigated in fibers. Though recent efforts to develop fiber-type in vivo biochemical sensors have brought successful results [9,10], the integrated electrochemical modalities are still not yet fully demonstrated, due to the lack of electrode materials which can satisfy both electrochemical sensing performance as well as thermomechanical restrictions imposed by the thermal drawing process. In recent studies, carbon-based polymer composites have been successfully integrated in the fibers [11], mainly for their enhanced electrical properties. Carbon is known for its stable performance in electrochemistry [12], and is widely used for electrochemical sensing, energy storage, or harvesting applications. To enable in-fiber electrochemistry, it is necessary to develop a functional carbon-based composite with enhanced electrochemical properties as well as consistent thermomechanical behaviors.

A high filler ratio is necessary for composites with submicron-size fillers [13] to facilitate sufficient bulk conductivity. This poses significant challenges to material processing and later thermal drawing, due to the increased viscosity. Furthermore, the homogeneous dispersion of loading particles becomes difficult due to the filler aggregation resulting from poor dispersion. In addition, the weak interfacial adhesion between the filler and the host matrix leads to both material degradation as well as mechanical failure in long-term use. Thus, it is crucial to design composites with an appropriate filler ratio of functional materials that possesses both easy processability, similar to the intrinsic polymer matrix, and enhanced functionality as a result of the interaction of the host matrix and the active fillers.

Among various conductive fillers, CNTs are chosen here for their large aspect ratio and ultra-high specific surface area, which are gifted with excellent mechanical, electrical, and electrocatalytic properties [13,14]. In particular, high electron transfer rate at the surface of composite with a higher loading of the CNTs is expected in the electrochemical measurements. CNT-based composites have been applied in electrochemical sensing, both as surface modifiers for conventional electrodes or as electrodes themselves [12,15]. However, homogeneous dispersion of nanoscale CNTs into the host polymer matrix is challenging due the agglomeration of CNTs as results of the Van der Waals interactions. It further imposes difficulties in achieving thermomechanical compatibility for the fiber drawing [16].

In this work, we investigated hybrid filler nanocomposites, containing both carbon nanotubes (CNTs) and carbon black (CB) to attain enhanced electrophysical and electrochemical properties and to explore their applicability in fibers for electrochemical sensing. The carbon-black-impregnated polyethylene (CPE) is chosen as the base polymetric material [2,11,17], we further developed hybrid nanocomposites via loading 3.8 wt%, 5 wt%, 7.5 wt%, and 10 wt% CNTs into the CPE based on the melting extrusion method. The series of nanocomposites have homogeneous dispersion of CNTs into the CPE. We further evaluated the electrical, thermal, and thermomechanical properties of the nanocomposites. The results showed the enhanced electrophysical characteristics and compatible viscoelastic properties with the thermal drawing process. Later, we screened compatible encapsulating thermoplastics and successfully fabricated CNT-CB hybrid nanocomposite fibers, which resulted in hundreds of meters of microelectrode fibers. A detailed characterization of their electrochemical sensitivity, detection limit in nanomolar scale, and long-term stability was performed. In particular, the surface electron transfer rate at the hybrid composite fiber was characterized via cyclic voltammetry in analytic solutions of ferrocenemethanol (FcMeOH), a standardized analyte in assessing the electrochemical properties of the sensors. Later, we demonstrated its proof-of-principle use in detecting electroactive biomolecules. Here, the dopamine (DA) was chosen as DA is known for its electrochemical profile [18,19] and physiological significance, such as its strong involvement in mediating our learning behaviors and motivations [20]. We have shown that down to 10 nM of DA can be reliably recorded via our fiber in in vitro settings without requiring any surface enhancement. In addition, we carried out the long-term experiments and incubated the fiber sensors in settings which mimic the in vivo or sweat environment with regulated temperature and ion composition. The sensitivity change over months was evaluated, which showed that there were no clear signs of sensing degradation in CNT-reinforced nanocomposite fibers over one month, whereas the pure carbon black based ones reached its lifetime at 2–3 weeks due to poor interfacial interactions of the nanofiller with the host polymer matrix.

In addition to the electrochemically active molecules, we also investigated our fiber-based microelectrode as an ion-selective electrode to detect specific ion concentrations. Here we focused on Na${}^{+}$ ion concentrations, which is important for sweat analysis in wearable devices [21]. For this, we functionalized our CNT hybrid composite fiber with ion sensitive membranes (ISM) that consist of ionophores and ion exchangers within poly(vinyl chloride) (PVC). We were able to selectively sense Na${}^{+}$ concentration with a relatively high average sensitivity of 49.48 ± 8.82 mV/decades, which shows no response to K${}^{+}$ ions.

The newly developed CNT hybrid nanocomposite materials and the nanocomposite fibers have shown excellent performance in electrochemical sensing to detect electrochemically active and inactive molecules. Further functionalization with other bioreceptors [22] will enable ultra-specific and sensitive detections of different compounds in the sweat or other body fluids.

## 2. Experimental Section

### 2.1. Synthesis of CNT Composites

The preparation of CNT-composites involves two major steps, consisting of dispersing CNT-loaded low density polyethylene (LDPE) master patches (PLASTICYL™ LDPE2001, Nanocyl SA, Sambreville, Belgium) into commercially available CPE (GoodFellow). Here MWCNT loaded LDPE was chosen due to its easy processiblity and high stability to prevent electrode fouling during electrode fabrication. As a first step, CPE films were cut, individually weighed, and placed on Teflon film. The CNT master patch pellets were also weighed and placed on the CPE sheets, and the amount of CNT master patch pellets were adjusted to reach the objective loading weight content of 3.8%, 5%, 7.5%, and 10%. Then, the CPE sheets with CNT pellets were pressed in a hot press (Lauffer Pressen UVL 5.0, Maschinenfabrik Lauffer GmbH & Co.KG, Horb am Neckar, Germany) at 200 °C and 50 N/cm${}^{2}$ for 15 min to embed the CNT pellets into CPE. One-step pressing was not able to achieve sufficient mixing; thus, we chopped the pressed film into small pellets and pressed again at 200 °C and 50 N/cm${}^{2}$ for 15 min.

**Figure 1:**
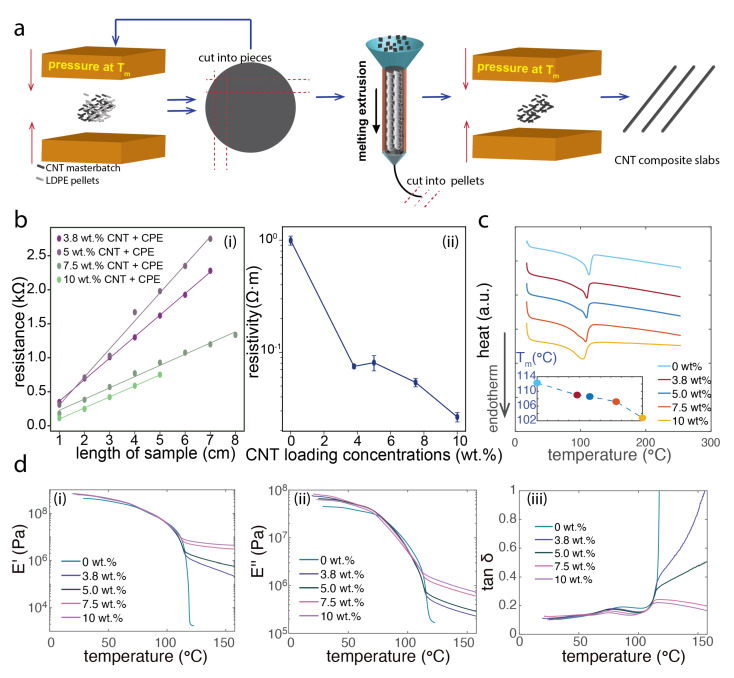
The composite synthesis with hybrid fillers (carbon nanotubes (CNTs) and carbon blacks (CBs)) and their detailed electrical, thermal and thermomechanical characterizations. (**a**) A schematic of synthesis process of the polymer composite with CNTs and CBs. (**b**) The electrical characterization of the bulk polymer composite showing homogeneous dispersion in (**i**) and the resistivity with a higher loading of CNTs. (**c**) Thermal analysis of crystallization and melting behavior of the composites. (**d**) Dynamic mechanical properties of the composites.

The obtained sheet was then chopped into about 2 mm × 2 mm pellets. They were fed into a twin-screw micro-extruder (DSM Micro 5) for melting extrusion in order to achieve a homogeneous dispersion of the CNTs inside of CPE. All sections of the extruder were preheated at 240 °C, and the screw was run at a speed of 100 rpm and extrusion was performed in inert nitrogen gas. The obtained extruded filament was later pressed again into a disk as composite electrode materials (Figure 1a).

### 2.2. Fiber Fabrication

The polyetherimide (Ultem) was initially chosen as the cladding material because of its high glass transition temperature (215 °C). The CNT composite electrode was machined into a slab with dimensions of 2 mm × 1 mm. A slot with the same cross-sectional size with slightly longer length, which was intended for the tolerance of the thermal expansion, was machined on the PEI slab with dimensions of 23 mm × 6 mm × 25 cm. The CNT composite electrode was inserted into the slot of PEI slab; another PEI slab was placed on top of it and consolidated as a dielectric layer.

**Figure 2:**
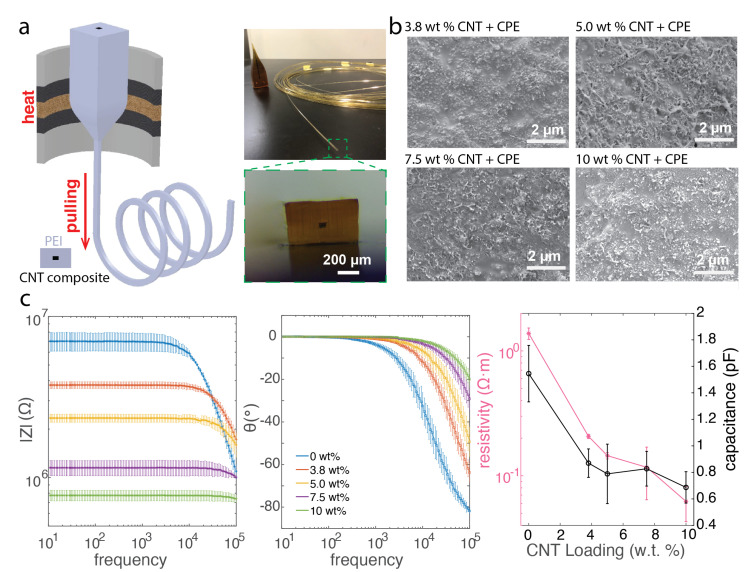
The CNT nanocomposite fibers fabricated via the thermal drawing process and their electrical characterizations. (**a**) A schematic of the thermal drawing process and photo of a fiber with its cross−section. (**b**) The scanning electron microscope images of the CNT composites with different loadings. (**c**) The electrical impedance analysis of the thermally drawn CNT composite fibers. The electrical impedance measurements were performed across different samples (N = 4).

The obtained preform had an overall size with dimensions of 23 mm × 12 mm × 25 cm and then was drawn at temperatures of 210 °C (top), 350 °C (middle), and 110 °C (bottom) into a fiber with hundreds-meter long in length using a custom-built fiber drawing tower (Figure 2a).

Similar procedures were done with fibers made of polycarbonate (PC) cladding, where only 0 wt%, 3.8 wt%, and 5 wt% CNT loaded composites were compatible due to a lower glass transition temperature (Tg: 145 °C).

### 2.3. Electrical Characterization

Different loadings of CNT composite slabs prior to the thermal drawing, which had a cross-section of about 0.52 mm × 2.0 mm and length from 10 cm to 1 cm, were prepared by low-end machining. The DC resistance was determined by the source meter where the silver paint (SPI supplies) was applied at both ends of the composite slab to ensure the minimal influence from the contact resistance. Then the composite slab was cut with a step of 1 cm and corresponding resistance was measured. From the results of resistance versus length, the dispersion quality of the composite can be confirmed. Later the resistivity of CNT composite before the thermal drawing was calculated by following the Pouillet’s law, given the known length and area (Figure 1b).

To obtain the electrical impedance, the thermally drawn fiber with CNT composite was cut into sections with the length of 3 cm, and the cross-sectional size of the CNT composite electrode was measured under an optical microscope. Then, conductive silver paint (SPI supplies) was applied on both ends to improve the surface contact and later was connected to copper wire to facilitate the electrical connection. Electrical impedance measurement was then performed using a potentiostat (Gamry Interface 1010E, Gamry Instruments Inc., Warminster, PA, USA) in the impedance analyzer mode between both ends of the fiber. A sinusoidal voltage with amplitude of 10 mV in the frequency range from 10 Hz to 100 kHz was applied between the two ends of the fiber, and the resulting alternating current was recorded, from which the electrical impedance of the CNT composite within the fiber was determined. Later, the electrical impedance results were analyzed via the Gamry software where we fitted the electrical impedance spectrum with the simple circuit model of resistance and capacitance in parallel. Again we could calculate the resistivity via following the Pouillet’s law, given the known length and area of the fiber. These analysis indicates both the resistive and the capacitive components in the fibers with CNT composite electrodes (Figure 2c).

### 2.4. Scanning Electron Microscopy Characterization

Freeze fracture in liquid nitrogen was used to prepare samples of CNT composite after the thermal drawing process. A thin gold layer or a thin palladium/platinum layer was deposited on the fiber cross-section prior to the scanning electron miscroscope (SEM) (HITACHI SU70, Hitachi High-Tech Corporation, Tokyo, Japan) observation (Figure 2b).

### 2.5. Thermal Characterization

Differential scanning calorimetry (Thermo plus EVO2 DSC8231, Rigaku Corporation, Tokyo, Japan) technique was used to characterize the thermal properties of the newly developed CNT composites. Samples of CNT composite were prepared as granules in a few mg weight, and corresponding aluminum oxide powder with the same weight was chosen as reference materials. The heating and cooling cycle from 20 °C to 250 °C with a rate of 10 °C/min was repeated twice to eliminate the thermal memory of the composite via the 1st cycle. The data of heat flow at the second cycle was analyzed to evaluate the microstructures in the nanocomposite (Figure 1c).

### 2.6. Dynamic Mechanical Analysis

Dynamic mechanical analysis (DMA) technique was used here to characterize the viscoelastic behavior of the CNT composites and cladding materials during the heating process. The CNT composite samples with size about 1 mm × 10 mm × 15 mm were prepared and mounted via the clap in the DMA machine (HITACHI DMA7100, Hitachi High-Tech Corporation, Tokyo, Japan). The temperature range was set from 20 °C to 160 °C with a heating rate of 2 °C/min for CNT loaded nanocomposites. The frequency of the stress was set as 1 Hz with an amplitude of 50 mN. From the DMA analysis, with the information of the storage modulus (E${}^{\prime }$) and loss modulus (E${}^{\prime \prime }$) during the heating process, we can assess the suitable temperature zone and corresponding the needed stress during the thermal drawing process. Ideally, for materials that are thermal drawing compatible, the suitable viscosity during the heating is preferred. Based on this, we can screen the encapsulating polymers for its compatibility for CNT composites as the cladding material. In addition to E${}^{\prime }$ and E${}^{\prime \prime }$, the loss factor tan(δ) can provide more information about the transition temperature of the composite material as well as insights of viscosity or elasticity dominated behavior of the CNT composites with CNT and polymer matrix interactions during heating (Figure 1d).

### 2.7. Cyclic Voltammetry

**Figure 3:**
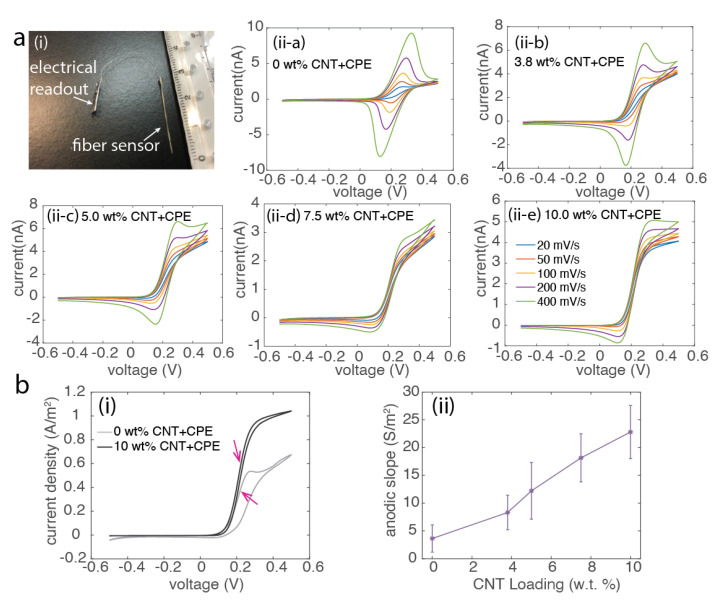
The electrochemical characterization of the CNT composite fibers. (**a**) Photograph of the fiber-based sensor in (**i**). (**ii**) The representative cyclic voltammograms (CV) of CNT nanocomposite fibers with varied scanning rates of 20 mV/s, 50 mV/s, 100 mV/s, 200 mV/s, and 400 mV/s, performed in 1 mM FcMeOH solutions. (**b**) The conductance per area of the electrode surface calculated based on the slope of the CV normalized by the electrode area in (**ii**). The representative CV curves obtained at 20 mV/s, a speed that all loadings of CNT composite fiber can achieve steady state. Measurements were performed across different samples (N ≥ 4).

The electrochemical performance of the CNT composite fibers were first characterized in 1 mM ferrocenemethanol (FcMeOH) dissolved in phosphate buffered saline solutions (PBS). The cyclic voltammetry measurements were performed using a potentiostat (Gamry Interface 1010E, Gamry Instruments Inc., Warminster, PA, USA), adopting the schemes of three-electrode configuration. The samples are connected to the working electrode, a glass-type Ag/AgCl reference (3M KCl, Basi) was used as the reference electrode, and a Pt wire was used as a counter electrode. The scanning rate of the cyclic voltammetry for FcMeOH solution was set from −0.5 V to 0.5 V with the varied scanning speed of 20 mV/s, 50 mV/s, 100 mV/s, 200 mV/s and 400 mV/s. From the CV results, the electrochemical reaction speed at the surface of the composite fiber was characterized via the slope of rising of anodic current at scanning speed of 20 mV/s (Figure 3).

### 2.8. Amperometric Measurement

**Figure 4:**
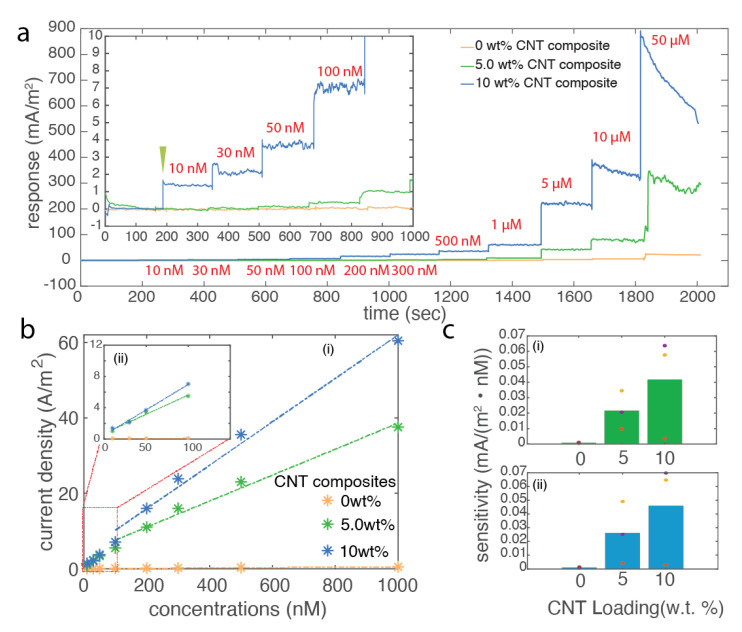
The DA sensing characterization of the CNT composite fiber. (**a**) Representative amperometry measurements of CNT composite fibers with DA sensing from 10 nM to 50 μM. (**b**) The relationship of the averaged current density versus concentrations and the linear fitting of the response in different concentration ranges in (**i**) and (**ii**). (**c**) The calculated sensitivity of CNT composite fibers with different loading. Individual measurement is color coded. Concentration range: (**i**) 100 nM to 1 μM and (**ii**) 10 to 100 nM.

CNT composite fibers were then used to detect the physiological component, DA in PBS (pH = 7.4) via the amperometric detection method. The measurement was performed using the potentiostat (Gamry Interface 1010E, Gamry Instruments Inc., Warminster, PA, USA), adopting both schemes of three-electrode and two-electrode configurations in 1× PBS(−). The oxidation potential of DA 0.6 V was applied between the reference electrode and the working electrode, then the current was collected between the working electrode and counter electrode. Prior to adding the DA, the baseline current response of 20 mL of PBS solution was stabilized for 5 min, then DA was added into the measurement solution. The concentrations of DA in the measurement solution were adjusted to 10 nM, 30 nM, 50 nM, 100 nM, 200 nM, 300 nM, 500 nM, 1 μM, 5 μM, 10 μM, and 50 μM sequentially and constantly mixed via a magnetic stirrer at the bottom. The period of each step was fixed at 3 min to ensure the full oxidation of the DA in the solution, then from the recorded current, it is possible to deduct the DA concentrations. The cyclic voltammetry measurements were also performed prior to and after the amperometric measurement of DA to confirm the success detection of DA in both amperometry and cyclic voltammetry (Figure 4).

### 2.9. Long-Term Evaluation of CNT Composite Fibers in Electrochemistry

**Figure 5:**
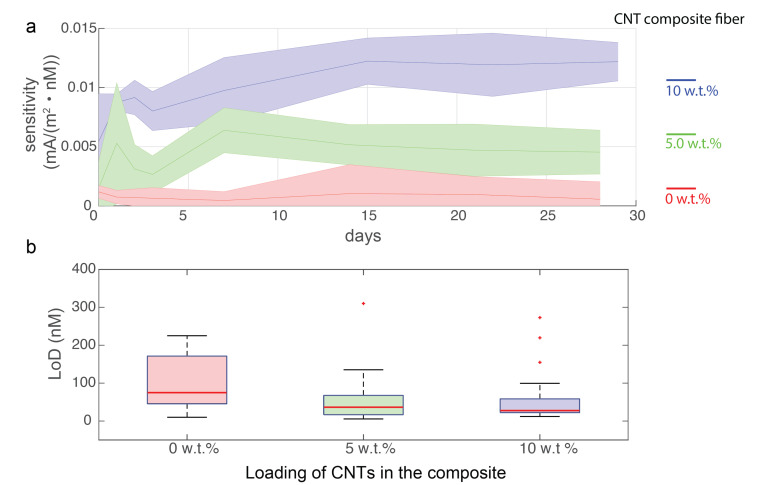
The long-term evaluations of the sensing performance (1 month) of the CNT nanocomposite fibers. (**a**) The sensitivity evolvement of the CNT nanocomposite fibers with different loadings. Different groups of CNT-based nanocomposite fibers are color coded. The solid line indicates the median of the sensitivity and the shaded area represents the standard deviation across samples. (N = 4 for 5, 10 wt% samples and N = 3 for 0 wt% samples) (**b**) The whisker plot of the low detection limit (LoD) calculated from amperometric data across fiber groups during the long-term evaluation. The median LoD for 0 wt%, 5 wt%, and 10 wt% are 75.0 nM, 36.4 nM, and 27.6 nM, respectively. The interquartile ranges (IQR) are 125.96 nM, 51.1 nM, and 36.4 nM, respectively.

CNT composite fibers with length of 3 cm were cut, the devices (N > 3) were prepared via connecting to copper wire using silver paste. They were then fixed via the 5 min epoxy to protect the connectorization parts. The proper functioning of the devices was first confirmed via cyclic voltammetry measurement. To mimic the in vivo implantation environment or in vitro sweating condition in close contact with skin, the devices were soaked into 1 × PBS solutions and stored in the dark at 37 °C. Then, the long-term stability of the device was evaluated via amperometric measurement of DA as in the aforementioned methods with narrower DA concentrations from 100 nM to 10 μM, where the CNT composite fiber exhibits linear sensing response. The series of amperometric measurements was performed at day 0, 1, 2, 3 and week 1, week 2, and week 4 to determine the long-term sensitivity changes of the CNT composite fiber as a microelectrode for biochemical sensing (Figure 5).

### 2.10. ISM Solutions and Coating on Microelectrode-Fibers

ISM solutions consisting of 330 mg (33% by weight) of PVC (high molecular weight, Selectophore grade, Sigma-Aldrich, St. Louis, MO, USA), 661 mg (66.1%) of DOS (041-18643, FUJIFILM Wako Pure Chemical Corporation, Osaka, Japan), 7 mg (0.7%) of sodium ionophore X (Selectophore grade, Sigma-Aldrich), and 2 mg (0.2%) of potassium tetrakis((4-chlorophenyl)- borate (purity ≥ 98.0%, Selectophore grade, Sigma-Aldrich) were added to 12.5 mL of tetrahydrofuran (THF; purity ≥ 99.9%, FUJIFILM Wako Pure Chemical Corporation, Osaka, Japan) [23]. The mixture was stirred until a clear solution was obtained with a concentration of 0.08 g/mL.

We prepared devices with 10 wt% CNT composite fiber of 3 cm in length, then we functionalized the fibers with dip coating. The tip of the fiber was immersed in to the ISM solutions for 5 s and allowed to dry for 60 s, this procedure was repeated 20 times. The ISM functionalized fiber devices were allowed to dry overnight. Then, they were conditioned overnight in the 100 mM NaCl solutions (50 mM Tris-HCl, pH = 7.8, FUJIFILM Wako Pure Chemical Corporation, Osaka, Japan). Before the measurement, the ISM functionalized fiber devices were washed in deionized(DI) water for 15 min, and were then ready to be evaluated as ISE fibers.

### 2.11. Characterization of Ion Sensitive CNT-Composite Fiber (ISE Fiber)

The continuous open circuit potential (OCP) measurements were performed using a potentiostat (Interface 1010E, Gamry Instruments) in a two electrode configuration, where no current follows. The ISE fibers were dipped in the measurement solution (50 mM Tris-HCl, pH = 7.8, FUJIFILM Wako Pure Chemical Corporation, Osaka Japan) while NaCl concentrations were increased by adding concentrated NaCl solutions or NaCl particles to reach 1 μM, 10 μM, 100 μM, 1 mM, 10 mM, 100 mM, 1 M. At each step, the solution was allowed to stabilize for 5 min.

### 2.12. Data Analysis

We prepared the customized MATLAB scripts to perform series analysis of data including CV measurements to characterize the electron transfer properties at the electrode surface, amperometric measurements to determine the sensitivity of the CNT composite fibers, and the OCP measurements to evaluated the ISM functionalized fiber devices in sensind Na${}^{+}$ ions.

The rising of the anodic current density was calculated by normalization of the area of the electrode, then the slope was calculated by linear regression fitting, which gives the averaged conductance of the electrode surface.

The amperometric response was normalized first by the electrode area. Then, a smoothing filter with average across 5 s was applied to the amperometric data. Afterwards, the baseline was calculated as the average of 2 min at 30 s prior to DA addition. Later, the current response of DA was calculated as the average of 2 min at 30 s post DA addition.

For long-term evaluation where the current density response versus DA concentration exhibits a linear response, such that the sensitivity was calculated based on the linear regression of both parameters, statistical analysis was performed across all measured samples by taking the median and standard deviation of their derived sensitivities.

Finally, the low detection limit (LoD) of the analyte concentration was determined based on 3 times the blank baseline current. Its statistical analysis across samples and measurements over the days was summarized via the whisker plot to describe the centre and spread of the distribution of LoDs.

The OCP response of ISE fibers was calculated as the average of 2 min at 30 s after increasing the Na${}^{+}$ concentrations, and the sensitivity was calculated based on the linear regression.

## 3. Results and Discussion

### 3.1. CNT Composite Materials

The fabrication of the CNT hybrid composite materials involves hot pressing and melt extrusion of the composite materials (Figure 1a). First, to characterize the dispersion quality of the CNTs within the composites, we used the electrical resistance as an indirect measure of the homogeneity. The results in Figure 1b, where the resistance of the bulk composite slabs is proportional to the length, show that the CNT dispersion within the composite was macroscopically homogeneous in the functional level. We further characterized the resistivity change with the increased loading of CNTs, where a drop of resistivity was observed as expected. Here, the base material CPE has a concentration of carbon black already exceeding the percolation threshold, so the observed resistivity change with hybrid loading of CNT is within two orders of magnitude. Both the electrical behaviors and the thermal characteristics were significantly altered (Figure 1c). We observed a decrease in melting temperature as well as a broadening of the endothermic peak with the increased loading of CNTs. These are because of the influence of the loading CNTs on the microstructure of the nanocomposites. With a higher loading of CNTs, the crystalline structure of the polyethylene (PE) gets smaller, which requires less energy to break upon heating, therefore we observed a decrease of $T_{m}$. The size distribution of the crystalline domains of PE also get wider as a result of the interactions of CNTs with the polymer chains; thus, we observed a wider endothermic peak. To evaluate the compatibility with the thermal drawing process, we needed to go beyond the melting temperature values and examine the flow behavior of the composites during the heating process. Here, we adopted the dynamic mechanical analysis method in Figure 1d to gain deeper insights of the microstructure evolution and viscoelastic behavior of the CNT composites. Without loading of CNTs, the CPE exhibits a fast drop of storage modulus (E${}^{\prime }$) of about 5 orders of magnitude and relatively slow drop of loss modulus (E${}^{\prime \prime }$) of about 3 orders of magnitude when the material is heated above its melting temperature. Because the crystalline structure within PE is still intact, it is highly viscous upon melt-flowing. Adding CNTs significantly alters the visco-elastic behaviors and the materials enter a rubbery state upon heating, as a result of the enhanced interactions of the CNTs with the polymer chains. More interestingly, with 3.8 wt% and 5 wt% loading of CNTs, the composites exhibit more viscous behaviors above $T_{m}$. The increased loading of CNTs within the nanocomposites, such as 7.5 wt% and 10 wt%, turns materials into a more elastic state above $T_{m}$, as manifested by the tanδ data, which indicates that they require a higher energy to deform during the thermal drawing process.

### 3.2. CNT Composite Fibers

Based on the visco-elastic behaviors of the CNT composite materials, we first chose polyetherimide (PEI) (Tg 215 °C) as the encapsulating polymer for drawing all sets of the CNT composites-based fibers in the same conditions (Figure 2a and Supplementary Figures S1 and S2). Then we examined the structural properties of nanocomposites after the thermal drawing by scanning electron microscope. Despite some microscaleCNT clusters as a result of the interactions of CNTs and polymer matrix, there is no mesoscale CNT agglomeration across samples. In addition, in nanocomposite fibers with a higher loading of CNTs, there are observably more CNTs being exposed at the fiber surface as shown in Figure 2b and Supplementary Figure S3. The electrical impedance spectrogram analysis (Figure 2c) of the thermally drawn fibers with different loadings of CNTs reveals that, at the functional level, the higher loading of CNT leads to an enhanced electrical conductivity which is consistent with the DC resistance measurement in the bulk nanocomposites (Figure 1b). In addition, the capacitive behavior existing in the dielectrical polymer composites was significantly reduced as CNTs, together with CBs, form a functional network to bridge the conductive domains and reduce the capacitance. The resistivity of the CNT nanocomposites before and after the thermal drawing process remain similar, which indicates that the CNTs within the nanocomposites maintained their random orientation without aligning to the extension direction during fiber drawing.

### 3.3. Electrochemical Characterization

The CNT-based nanocomposite fibers exhibit enhanced electrical behaviors and there are considerable amounts of CNTs being exposed at the fiber surface, thus an enhanced electron transfer rate at the surface is expected for electrochemical sensing applications.

To evaluate this, here, we adopted the cyclic votammetry (CV) technique (Figure 3a), where a cycling of potential is applied to the working electrode and the resulting current from the oxidation in the forward scan and reduction in the reverse scan is recorded. We carried out the CV measurements in a solution of the standard analyte, ferrocenemethanol (FcMeOH). FcMeOH was chosen because it can undergo a reversible one-electron redox reaction via potential sweepings. As CNT nanocomposite fibers have the electrode in the micrometer scale, so the characteristic time, related to the scanning rate of potentials, significantly influences the diffusion profile of the microelectrode—the CNT-based nanocomposite fibers. There exist two dominant parameters—either the mass transport or the electron transfer rate that determines the shape of the voltammogram upon varying the scanning rate.

The current transient following a potential step is defined by

(1) $$i\left(t\right)=\frac{nFAD^{1/2}C^{*}}{\pi^{1/2}t^{1/2}}+\frac{nFADC^{*}}{r}$$

*n*: number of transfered electrons
*F*: Faraday constant
*A*: electrode area (effective)
*D*: diffusion coefficient
$C^{*}$: concentration of analytes at equilibrium
*r*: electrode radius (effective)

which consists of both a time-dependent and a time-independent factor [24].

For the microelectrode, at a lower scan rate, the current response is primarily dominated by the radical diffusion, thus the mass transportation rate. Their CV profile exhibits a sigmoidal shape with their steady state current determined primarly by the time-independent factor in the Equation (1).

(2) $$i=\frac{nFADC^{*}}{r}=4\pi nFDC^{*}r$$

However, with the increased scanning rate, electron transfer rate overcomes the mass transport rate, the current transient can no longer achieve steady state. Both time-dependent and time-independent factors contribute to the current transient, thus CVs of nanocomposites fibers starts to have anodic and cathodic peaks, which are commonly observed at macro-electrodes.

We evaluated the electron transfer rate based on their CV profiles (Figure 3b). With scanning rate increasing from 20 mV/s to 400 mV/s, the samples with no or less CNT loaded composite materials were no longer able to achieve steady-state. While with a higher loading of CNTs, the sigmoidal shape was maintained even at CV performed at the high scanning rate of 400 mV/s. We further quantitatively examined the electron transfer rate at the electrode surface. At a slower scanning rate −20 mV/s, all the samples could reach their steady state as expected. The current at the steady state is determined by the diffusion factor of the species, but how fast the CV profile reaches its steady state is primarily determined by the electron transfer rate. We calculated the steepness of rising part of the anodic curve in Figure 3b(ii), which gives the conductance per area of the electrode surface. We observed an increase in the conductance of electrode surface for electrochemical reactions with a higher loading of CNTs, which indicates an enhanced electron transfer rate. So the electrochemical sensitivities of CNT nanocomposite fibers can be improved with a higher loading of CNTs, when developed as electrochemical sensors.

### 3.4. Electrochemical Sensing

Owing to the miniature size of the fiber microelectrode and long-term biocompabilities reported in the previous literatures [2,11], the CNT-based nanocomposite fibers represent excellent candidates for in vivo biochemical sensing applications, particularly to interface with the brain, as well as for smart textile applications to monitor physiological chemicals related to health. Here, in this study, we first chose the DA as an analyte, as it is an important neurotransmitter existing in our reward circuit in brain for mediating learning and motivations. We use the amperometry technique; a fixed voltage is applied to the working electrode, at which the DA can be oxidized, the oxidation current is recorded with the precise temporal resolution. Throughout this set of experiments, we could evaluate both the sensitivity and the practical low detection of the CNT-based nanocomposite fibers in in vitro settings. In Figure 4a, the amperometric data show a step increase in current with the addition of DA into the PBS solution. In particular, the 10wt% CNT composite fibers could detect concentrations as low as 10 nM, the step increase in current density was about 35.6 times higher than the noise level calculated as the root mean square of the current density fluctuation after adding DA. Such precise detection in low concentration is close to the in vivo concentrations. Then, we further quantified the current density change with concentrations of DA. As we examined a quite large range of the concentrations across almost four orders of magnitudes, the relationship between them exceeded the linear response. Thus, we calculated the sensitivity based on different concentration ranges, such as a physiological range of 100 nM to 1 μM, and low concentrations ranges from 10 nM to 100 nM (Figure 4b,c). With higher loading of CNTs, there is a significant increase in the sensitivities in both concentration ranges, thanks to the fast electron transfer rate offered by the CNTs within the composites. It is particularly useful for in vivo applications where an ultra-high sensitivity down to nanomolar or even picomolar range is needed.

### 3.5. Long-Term Evaluation

For applications such as in vivo chemical sensing or smart textiles for health monitoring, biosensors needs to be sufficiently stable for long-term use. Here we evaluated the sensitivity change with prolonged exposure to the temperature-regulated phosphate buffered saline (PBS) solutions, an environment that mimics the in-body or skin environment. We examined the DA sensitivity in the 100 nM to 10 μM range, where the relationship between the current response and concentration exhibits a linear relationship. The results (Figure 5) show that the CNT loaded nanocomposite fibers have prolonged stability in its sensing performance over 1 month immersion into the PBS(−) at 37 °C. It proves that there is no significant degradation of polymer composites; the surface electrochemistry of the electrode is well-maintained (Supplementary Figure S2). However, the nanocomposite fiber that does not contain CNTs began to fail at the 2–3 week time point, as a result of poor adhesions of the polymer matrix and the filler particles—carbon black in this case. The CNTs reinforce the interaction of the functional fillers with the polymer matrix and increase its functional and mechanical durability. In addition, the low detection limit (calculated as the concentration corresponding to three times the blank current) across different fiber groups were evaluated across the long-term evaluation. We found that nanocomposite fibers with higher loading of CNT have lower LoD, which is expected from the enhanced sensing performance by the loading of CNTs (Figure 5b). In addition, the fluctuation across the long-term evaluation in composite fibers with high loading of CNTs, such as 10 w.t% CNT composite fiber, is much smaller compared to the ones without or with less CNTs as indicated in Figure 5b with reduced IQR values. Thus our fiber holds great potential for long-term use where high stability in sensing performance is required, particularly, for in vivo applications which require correlating chemical releases with longitudinal behavioral studies.

### 3.6. Evaluation of the Na${}^{+}$ Sensing Performance

**Figure 6:**
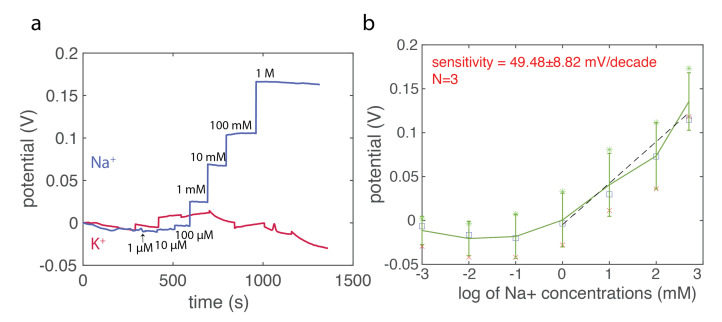
The Na${}^{+}$ sensing characterization of the ion-sensitive membrane (ISM) functionlized CNT composite fibers. (**a**) The representative continuous measurement of the ISM-functionalized fibers to Na${}^{+}$ ions (blue) and its selective response to the K${}^{+}$ ions (red). (**b**) The sensitivity calculation of the Na${}^{+}$ ion response across different devices (N = 3) with an average sensitivity of 49.48 ± 8.82 mV/decade. Different markers indicate individual devices with its measurement.

The conventional ion-sensitive electrodes(ISEs) are based on the liquid contact electrodes which consist of a metal electrode and the ISM, which are filled with an aqueous solution. As such configuration requires liquid containers, which imposes difficulties in designing wearable systems and further limits the miniaturization in sensors, the ISM-coated electrodes are being developed to address this. However, electrode materials are typically based on rigid metals, which makes it difficult to conform them with skin. In addition, the polymer-based ISMs have a relatively poor attachment to the metal, and a thin aqueous layer can form between the electrode and the ISM, which leads to instabilities in measurements. Therefore, it is interesting to study the performance of the ISM integrated with flexible CNT composite microelectrode fibers, which may offer new possibilities of ISE fibers in wearable devices for health monitoring. First we focused on the fiber tip functionlized with the ISM to show the proof-of-concept detection of specific ions, however, it can be further extended on the side of the fiber as well. Here, the continuous potentiometric measurement was adopted, the ISE fiber was dipped in Tris-buffer solutions (50 mM Tris HCl buffer, FUJIFILM Wako Pure Chemical Corporation), while NaCl concentrations were increased within the buffer solutions. Tris-buffer solutions were used here, as proton concentrations are regulated to eliminate their influence. The results (Figure 6) reveal the steady increase of the OCPs with the increase of the NaCl concentrations. In addition, it has no response to interferent K${}^{+}$ ions. We further calculated the linear sensitivity between 1 mM to 1 M across different devices with the average of 49.48 ± 8.82 mV/decade. It is close to the theoretical value of 59.2 mV/decades for a monovalent ion sensing at 25 °C based on the Nikolskii–Eisenman equation. It shows that our ISM-functionlized CNT composite fiber can work properly as an ISE fiber for specific ions, extending possibilities in applications such as the wearable devices or in vivo monitoring of ion flunctations.

## 4. Conclusions

In this work, we have developed a series of CNT-loaded nanocomposites and examined their electrical, thermal, and thermomechanical properties. We were able to incorporate these materials into fibers via the thermal drawing process to develop miniature fiber-based flexible electrochemical sensors. First, we evaluated the surface electron transfer capabilities via the cyclic voltammogram, from which we calculated the slope of the rising of the anodic current. We observed that the conductance per unit area increased with the increased loading of CNTs. These are direct evidence of the faster electron transfer rate achieved with higher loadings of CNTs. We then prepared the microelectrode sensor based on our composite fibers, and were able to detect the DA in in vitro settings down to 10 nM with the 10 wt% CNT composite fiber. Such results show that the CNT-modified electrodes exhibit ultra-high sensitivity without the need of additional steps for the surface functionalization. To further demonstrate its long-term structural and functional stability in either in vivo or in vitro settings, we evaluated the sensitivity and low detection limit change of the CNT composites fiber immersed into saline solutions over months. We show that over the course of one-month immersion into physiological solutions, the composite-based fibers maintained their stability in biochemical sensing, indicating the composite materials are mechanically intact and maintain their functional performance. Lastly, we combined the ISM on the CNT composite fibers to extend the sensing capability to electrochemically inactive molecules—ions. We were able to detect Na${}^{+}$ ions—an important biomarker in sweat indicating our health status, with a reliable sensitivity of 49.48 ± 8.82 mV/decade using our ISE fibers. In addition, there was no response to interferent ions such as K${}^{+}$.

The results obtained here demonstrate the possibilities of further developing the thermally-drawn fibers towards biochemical sensing applications for fundamental biological studies as well as healthcare applications. Currently, there is a trade-off between the thermocompatibility imposed by the thermal drawing process and the sensitivity enhanced by the functional filler loading. We need to further improve the composition, such as by introducing unique 2D materials—Mxene [25] and graphene [26], which have enriched surface chemistry. In addition, longitudinal functionalization of fibers can be realized via nano/micro-scale patterning technologies, such as laser micro-machining for multiplexed sensing. With the recent development of the convergence drawing technique, we can easily integrate reference electrode, counter electrode, as well as composite-material-based working electrode into fibers [10], from which we can develop the standalone miniature fiber-based all-in-one electrochemical detector. Such fiber-based bioelectronics hold great potentials for long-term chemical monitoring in vivo or in vitro, which will not only advance fundamental biological studies which require correlating in vivo chemical releases with longitudinal behavioral output, but also point-of-care applications where fibers are integrated into textiles for seamless health monitoring.

## Data Availability

The data reported in this work are available upon reasonable request to the corresponding author.

## Supplementary Materials

The following supporting information can be downloaded at: https://www.mdpi.com/article/10.3390/bios12080559/s1. Figure S1: The DMA analysis of polymer - polycarbonate (PC) and polyetherimide (PEI). Figure S2: The thermal drawing parameters of the CNT composite fibers with different loadings based on the PEI as claddings. Figure S3: The scanning electron microscope (SEM) analysis of the morphology and composition of the nanocomposites. Figure S4: The cyclic voltammograms of the fiber sensors with 10 wt% CNT nanocomposites (N = 4). Figure S5: The Na${}^{+}$ sensing characterization of the ion-sensitive membrane functionlized CNT composite fibers in the physiological range.

## References

[1] Yan W., Page A., Nguyen-Dang T., Qu Y., Sordo F., Wei L., Sorin F. (2019). Advanced multimaterial electronic and optoelectronic fibers and textiles. Adv. Mater..

[2] Park S., Guo Y., Jia X., Choe H.K., Grena B., Kang J., Park J., Lu C., Canales A., Chen R. (2017). One-step optogenetics with multifunctional flexible polymer fibers. Nat. Neurosci..

[3] Rein M., Favrod V.D., Hou C., Khudiyev T., Stolyarov A., Cox J., Chung C.C., Chhav C., Ellis M., Joannopoulos J. (2018). Diode fibres for fabric-based optical communications. Nature.

[4] Leber A., Page A.G., Yan D., Qu Y., Shadman S., Reis P., Sorin F. (2020). Compressible and Electrically Conducting Fibers for Large-Area Sensing of Pressures. Adv. Funct. Mater..

[5] Yan W., Richard I., Kurtuldu G., James N.D., Schiavone G., Squair J.W., Nguyen-Dang T., Gupta T.D., Qu Y., Cao J.D. (2020). Structured nanoscale metallic glass fibres with extreme aspect ratios. Nat. Nanotechnol..

[6] Keshavarz M., Wales D.J., Seichepine F., Abdelaziz M.E., Kassanos P., Li Q., Temelkuran B., Shen H., Yang G.Z. (2020). Induced neural stem cell differentiation on a drawn fiber scaffold—Toward peripheral nerve regeneration. Biomed. Mater..

[7] Koppes R.A., Park S., Hood T., Jia X., Poorheravi N.A., Achyuta A.H., Fink Y., Anikeeva P. (2016). Thermally drawn fibers as nerve guidance scaffolds. Biomaterials.

[8] Kimmel D.W., LeBlanc G., Meschievitz M.E., Cliffel D.E. (2012). Electrochemical sensors and biosensors. Anal. Chem..

[9] Guo Y., Werner C.F., Canales A., Yu L., Jia X., Anikeeva P., Yoshinobu T. (2020). Polymer-fiber-coupled field-effect sensors for label-free deep brain recordings. PLoS ONE.

[10] Guo Y., Werner C.F., Handa S., Wang M., Ohshiro T., Mushiake H., Yoshinobu T. (2021). Miniature multiplexed label-free pH probe in vivo. Biosens. Bioelectron..

[11] Guo Y., Jiang S., Grena B.J., Kimbrough I.F., Thompson E.G., Fink Y., Sontheimer H., Yoshinobu T., Jia X. (2017). Polymer composite with carbon nanofibers aligned during thermal drawing as a microelectrode for chronic neural interfaces. ACS Nano.

[12] Chen R., Li Y., Huo K., Chu P.K. (2013). Microelectrode arrays based on carbon nanomaterials: Emerging electrochemical sensors for biological and environmental applications. RSC Adv..

[13] Thostenson E.T., Ren Z., Chou T.W. (2001). Advances in the science and technology of carbon nanotubes and their composites: A review. Compos. Sci. Technol..

[14] Levchenko V. (2011). Structure and Electrophysical Properties of Nanocomposites Based On Thermoplastic Polymers and Carbon Nanotubes. Ph.D. Thesis.

[15] Wightman R.M. (2006). Probing cellular chemistry in biological systems with microelectrodes. Science.

[16] Suhr J., Zhang W., Ajayan P.M., Koratkar N.A. (2006). Temperature-activated interfacial friction damping in carbon nanotube polymer composites. Nano Lett..

[17] Ma P.C., Liu M.Y., Zhang H., Wang S.Q., Wang R., Wang K., Wong Y.K., Tang B.Z., Hong S.H., Paik K.W. (2009). Enhanced electrical conductivity of nanocomposites containing hybrid fillers of carbon nanotubes and carbon black. ACS Appl. Mater. Interfaces.

[18] Jackowska K., Krysinski P. (2013). New trends in the electrochemical sensing of dopamine. Anal. Bioanal. Chem..

[19] Abe H., Ino K., Li C.Z., Kanno Y., Inoue K.Y., Suda A., Kunikata R., Matsudaira M., Takahashi Y., Shiku H. (2015). Electrochemical imaging of dopamine release from three-dimensional-cultured PC12 cells using large-scale integration-based amperometric sensors. Anal. Chem..

[20] Wise R.A. (2004). Dopamine, learning and motivation. Nat. Rev. Neurosci..

[21] Chung M., Fortunato G., Radacsi N. (2019). Wearable flexible sweat sensors for healthcare monitoring: A review. J. R. Soc. Interface.

[22] Booth M.A., Gowers S.A., Hersey M., Samper I.C., Park S., Anikeeva P., Hashemi P., Stevens M.M., Boutelle M.G. (2021). Fiber-based electrochemical biosensors for monitoring pH and transient neurometabolic lactate. Anal. Chem..

[23] Roy S., David-Pur M., Hanein Y. (2017). Carbon nanotube-based ion selective sensors for wearable applications. ACS Appl. Mater. Interfaces.

[24] Zoski C.G. (2007). Handbook of Electrochemistry.

[25] Gogotsi Y., Anasori B. (2019). The rise of MXenes. ACS Nano.

[26] Kuila T., Bose S., Khanra P., Mishra A.K., Kim N.H., Lee J.H. (2011). Recent advances in graphene-based biosensors. Biosens. Bioelectron..

