# Supplementary material for: Thermally Drawn CNT-Based Hybrid Nanocomposite Fiber for Electrochemical Sensing
Source: Biosensors (Basel). 2022 Jul 24;12(8):559. doi: 10.3390/bios12080559 (PMC9394265; doi:10.3390/bios12080559)
Supplement: Supplementary file 1 [file biosensors-12-00559-s001.zip › biosensors-1827808-SI.pdf]

biosensors

MDPI

Article

Thermally drawn CNT-based hybrid nanocomposite fiber for electrochemical sensing

Rino Nishimoto[1], Yuichi Sato[2], Tomoki Saizaki[3], Mahiro Kubo[3], Jingxuan Wu[1], Mengyun Wang[1], Hiroya Abe[1], Inès Richard[4], Tatsuo Yoshinobu[1,5], Fabien Sorin[4] and Yuanyuan Guo[2,5,6,*]

1 Graduate School of Engineering, Tohoku University, Sendai, Japan, 980-8579
2 Frontier Research Institute for Interdisciplinary Sciences (FRIS), Tohoku University, Sendai, Japan, 980-0845
3 School of Engineering, Tohoku University, Sendai, Japan 980-8579
4 Institute of Materials, Ecole Polytechnique Fédérale de Lausanne (EPFL), Lausanne 1015, Switzerland
5 Graduate School of Biomedical Engineering, Tohoku University, Sendai, Japan, 980-8579
6 Graduate School of Medicine, Tohoku University, Sendai, Japan, 980-8575
* Correspondence: yyuanguo@fris.tohoku.ac.jp;

check for updates

Citation: Nishimoto, R.; Sato, Y.; Saizaki, T.; Kubo, M.; Wu, J.; Wang. M.; Abe, H.; Richard, I; Yoshinobu, T; Sorin, F; Guo, Yuanyuan Thermally drawn CNT-based hybrid nanocomposite fiber for electrochemical sensing. *Biosensors* **2022**, *12*, 559. https://doi.org/10.3390/bios12080559

Received: 6 July 2022
Accepted: 22 July 2022
Published: 24 July 2022

Publisher's Note: MDPI stays neutral with regard to jurisdictional claims in published maps and institutional affiliations.

Copyright: © 2022 by the authors. Licensee MDPI, Basel, Switzerland. This article is an open access article distributed under the terms and conditions of the Creative Commons Attribution (CC BY) license (https://creativecommons.org/licenses/by/4.0/).

List of Figures

1. **The DMA analysis of polymer - polycarbonate (PC) and polyetherimide (PEI). a.** The storage modulus, the loss modulus and tan(δ) of the PC from 20°C to 160°C. **b.** The storage modulus, the loss modulus and tan(δ) of the PEI from 20°C to 240°C. We identified that PC is compatible with nanocomposites with CNT loadings up to 5 wt% and PEI is compatible with nanocomposites with CNT loadings up to 10 wt%. 2
2. **The thermal drawing parameters of the CNT composite fibers with different loadings based on the PEI as claddings. a.** The drawing temperature, the long side of the cross-sectional length and the tension during the process of drawing PEI with 0 wt% loading of composites and with 3.8 wt, 5.0 wt, 7.5 wt and 10 wt % of CNT nanocomposites in **b-e**, respectively. 3
3. **The scanning electron microscope (SEM) analysis of the morphology and composition of the nanocomposites. a.** The SEM of the nanocomposite without CNT loadings. **b.** The detailed SEM of the nanocomposites with 3.8 wt, 5.0 wt, 7.5 wt and 10 wt % loading of CNTs. 4
4. **The cyclic voltammograms of the fiber sensors with 10 wt% CNT nanocomposites. (N=4)** The data were obtained across 4 different samples and the scanning rate varied from 20 mV/s to 400 mV/s. 5
5. **The Na^+ sensing characterization of the ion-sensitive membrane functionlized CNT composite fibers in the physiological range. a.** The continuous measurement of the ISM-functionalized fibers to Na^+ ions and its selective response to the K^+ ions. **b.** The sensitivity calculation of the Na^+ ion response, which exhibits an linearity from 1 mM with sensitivity of 53.35 mV/decades. 5

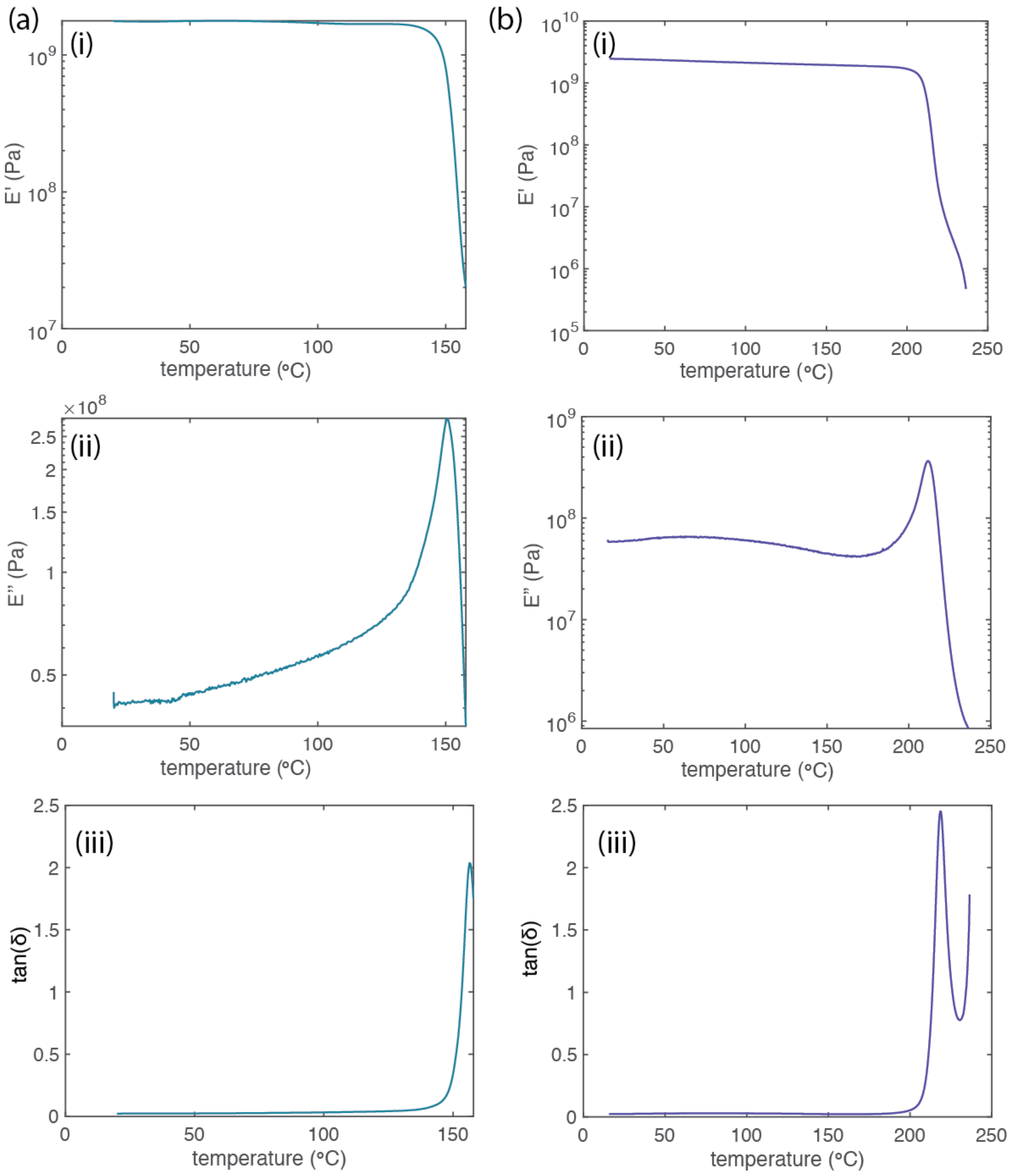

Figure S1. The DMA analysis of polymer - polycarbonate (PC) and polyetherimide (PEI). a. The storage modulus, the loss modulus and tan(δ) of the PC from 20°C to 160°C. **b.** The storage modulus, the loss modulus and tan(δ) of the PEI from 20°C to 240°C. We identified that PC is compatible with nanocomposites with CNT loadings up to 5 wt% and PEI is compatible with nanocomposites with CNT loadings up to 10 wt%.

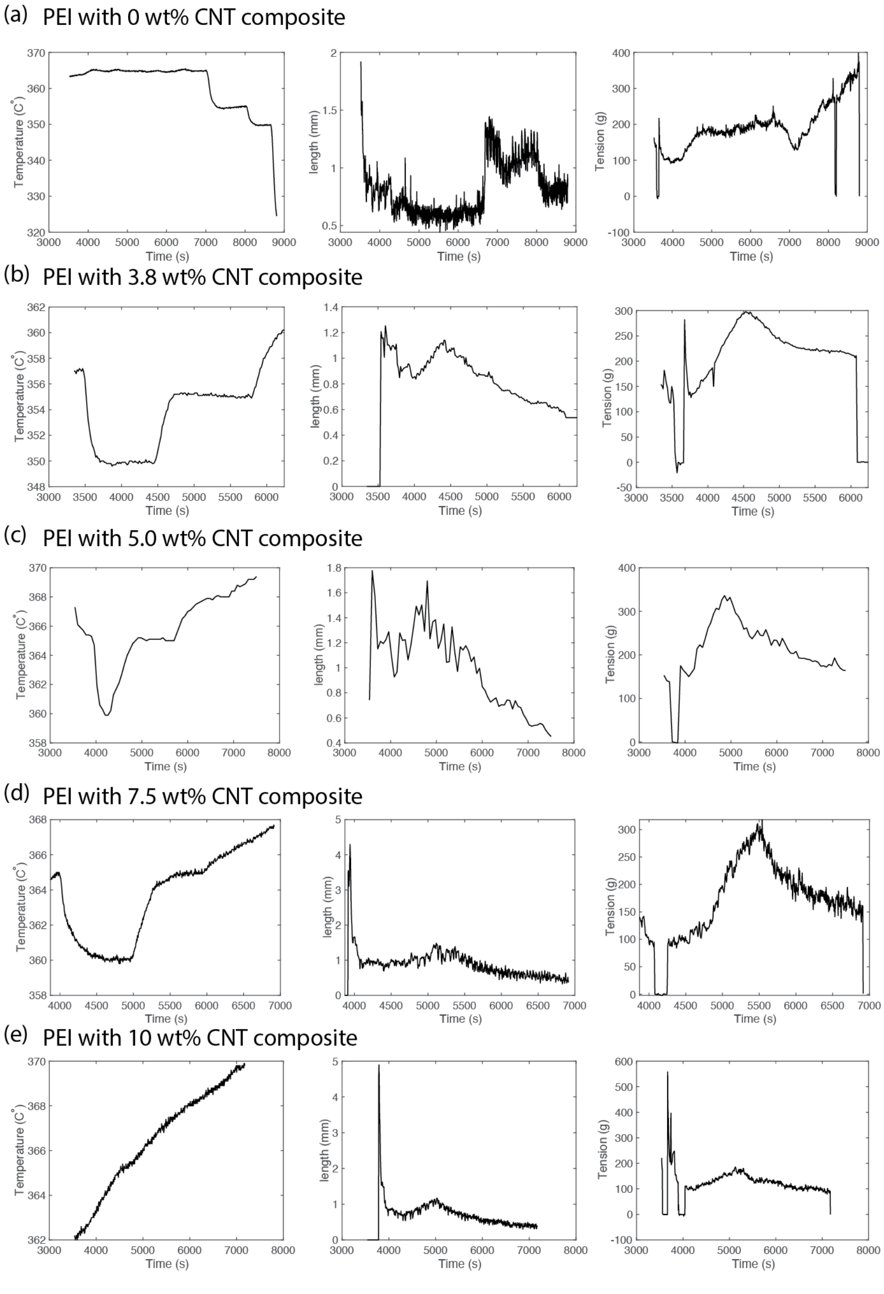

Figure S2. The thermal drawing parameters of the CNT composite fibers with different loadings based on the PEI as claddings. a. The drawing temperature, the long side of the cross-sectional length and the tension during the process of drawing PEI with 0 wt% loading of composites and with 3.8 wt, 5.0 wt, 7.5 wt and 10 wt % of CNT nanocomposites in **b-e**, respectively.

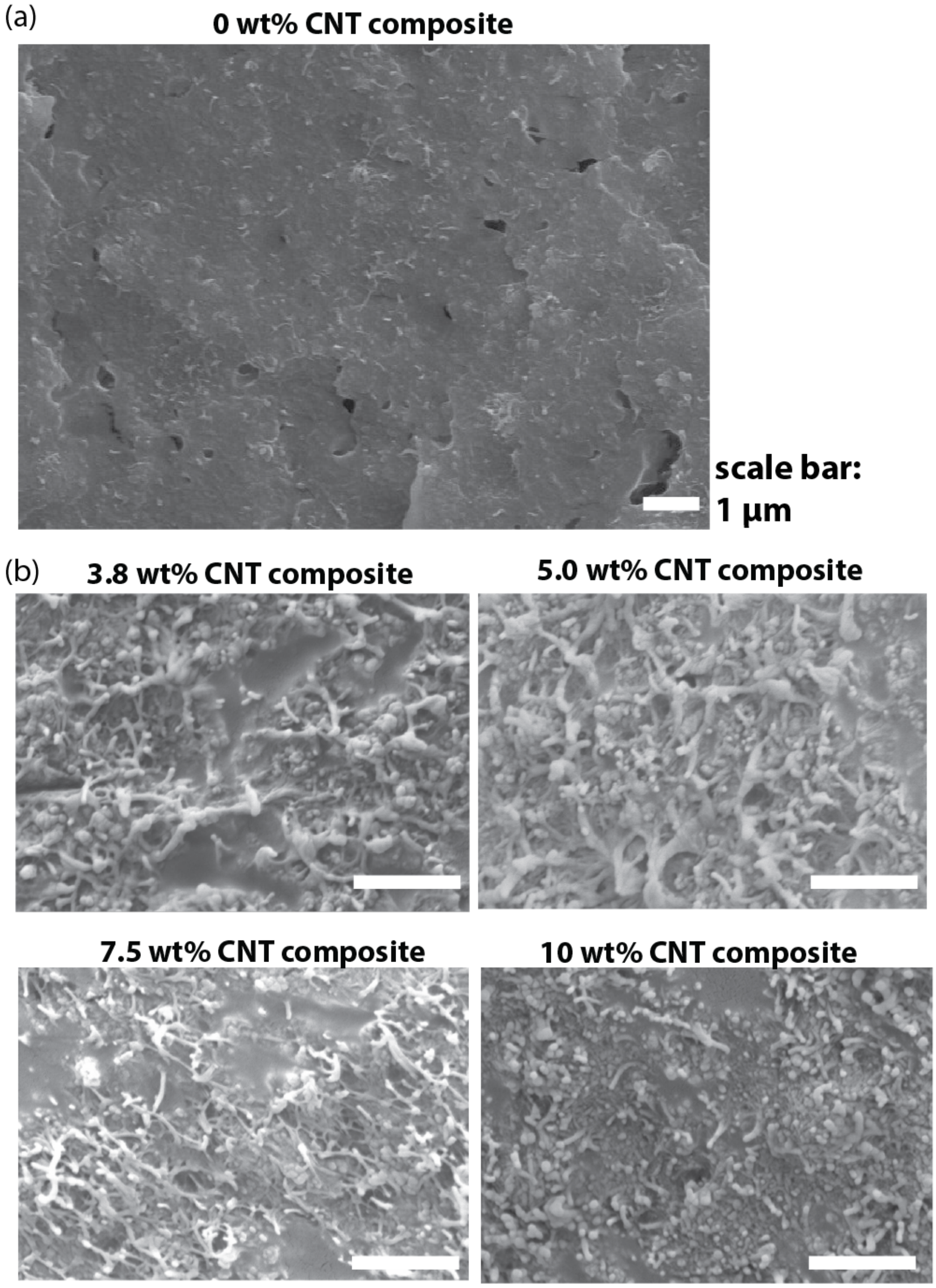

Figure S3. The scanning electron microscope (SEM) analysis of the morphology and composition of the nanocomposites. a. The SEM of the nanocomposite without CNT loadings. **b.** The detailed SEM of the nanocomposites with 3.8 wt, 5.0 wt, 7.5 wt and 10 wt % loading of CNTs.

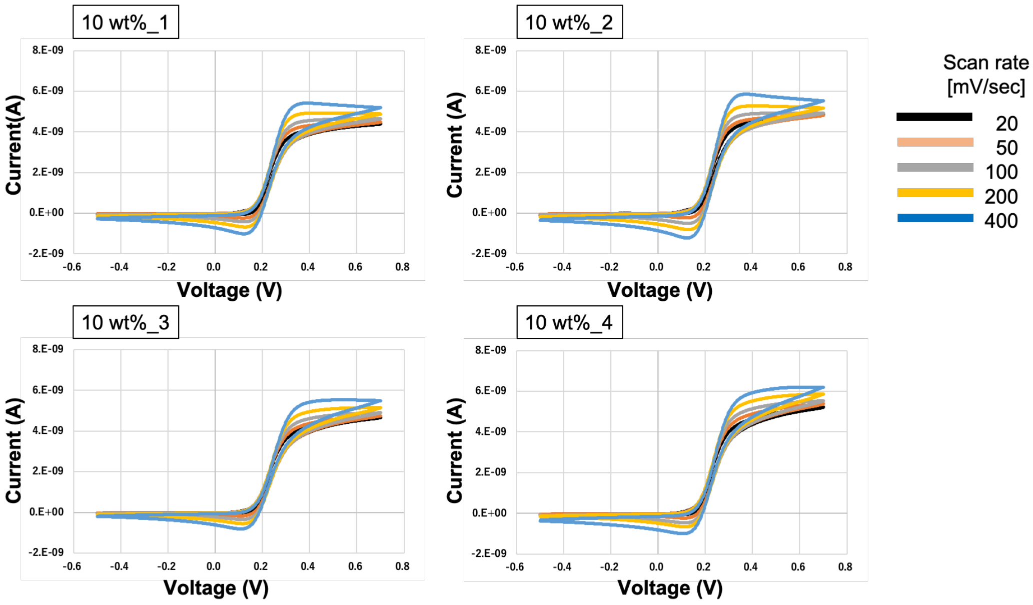

Figure S4. The cyclic voltammograms of the fiber sensors with 10 wt% CNT nanocomposites. (N=4) The data were obtained across 4 different samples and the scanning rate varied from 20 mV/s to 400 mV/s.

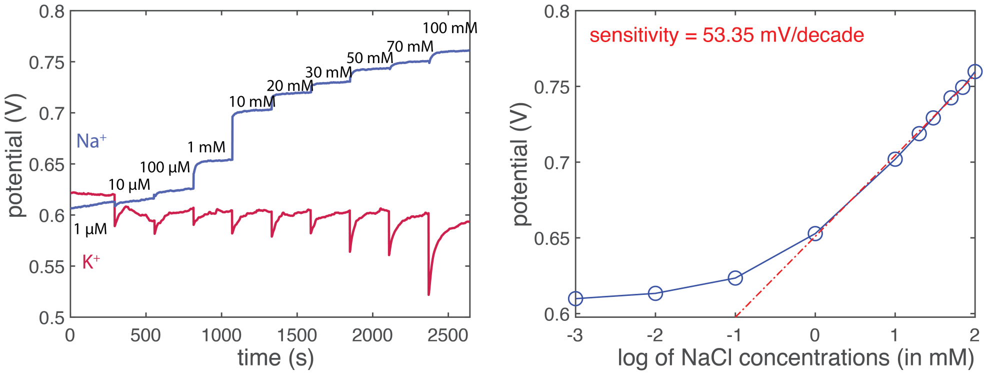

Figure S5. The Na^+ sensing characterization of the ion-sensitive membrane functionlized CNT composite fibers in the physiological range. a. The continuous measurement of the ISM-functionalized fibers to Na^+ ions and its selective response to the K^+ ions. **b.** The sensitivity calculation of the Na^+ ion response, which exhibits an linearity from 1 mM with sensitivity of 53.35 mV/decades.
